# A Prospective, Multicenter Analysis of Recurrence‐Free Survival After Sentinel Lymph Node Biopsy Decisions Influenced by the 31‐GEP

**DOI:** 10.1002/cam4.70839

**Published:** 2025-04-01

**Authors:** J. Michael Guenther, Andrew Ward, Brian J. Martin, Mark Cripe, Timothy Beard, Oliver Wisco, Rohit Sharma, Stanley P. Leong, Richard Essner, Joseph I. Clark, John Hamner, Brenda Sickle‐Santanello, Maki Yamamoto

**Affiliations:** ^1^ St Elizabeth Physicians General & Vascular Surgery Edgewood Kentucky USA; ^2^ University of Tennessee Medical Center Knoxville Tennessee USA; ^3^ Castle Biosciences Friendswood Texas USA; ^4^ OhioHealth Physician Group Columbus Ohio USA; ^5^ Summit Medical Group Bend Oregon USA; ^6^ Brown Dermatology Providence Rhode Island USA; ^7^ Marshfield Clinic Marshfield Wisconsin USA; ^8^ California Pacific Medical Center and Research Institute San Francisco California USA; ^9^ Melanoma and Cutaneous Oncology Research Program Saint John's Cancer Institute Santa Monica California USA; ^10^ Loyola University Medical Center Maywood Illinois USA; ^11^ University of Colorado Denver Colorado Springs Colorado USA; ^12^ Surgical Oncology Associates of Columbus Columbus Ohio USA; ^13^ School of Medicine University of California‐Irvine Orange California USA

## Abstract

**Background:**

Although most patients with cutaneous melanoma (CM) will have a negative sentinel lymph node biopsy (SLNB), up to 20%–30% of these patients will recur. The 31‐gene expression profile (31‐GEP) test has been prospectively validated to identify patients at low (Class 1A), intermediate (Class 1B/2A), and high (Class 2B) risk of SLN positivity and recurrence.

**Methods:**

DECIDE is a prospective, multicenter study to assess the effect of 31‐GEP testing on SLNB performance rates in patients with T1–T2 tumors considering SLNB and to study long‐term outcomes. Here, we assessed outcomes in patients with a Class 1A 31‐GEP result (*n* = 130).

**Results:**

Of Class 1A patients, 63 had an SLNB, with a 3.2% SLN positivity rate (2/63). No Class 1A patients, regardless of SLN status, experienced a recurrence (2‐year median follow‐up).

**Conclusions:**

These results are consistent with previous studies that showed the 31‐GEP can identify patients at low risk of SLN positivity and recurrence.

## 1 Introduction

Sentinel lymph node biopsy (SLNB) for cutaneous melanoma (CM) is considered prognostic for the risk of melanoma recurrence and patient survival [1]. Guidelines for SLNB suggest avoiding the procedure if the risk of a positive node is < 5%, considering SLNB if the risk is 5%–10%, and offering SLNB if the risk is > 10% [2]. Traditionally, guidelines for SLNB recommendations are based on a patient's predicted risk of SLN positivity using only clinicopathologic features, including Breslow thickness, ulceration, age, mitotic rate, anatomic location, and lymphovascular invasion [2]. However, relying upon these traditional clinicopathologic features results in up to 88% of SLNB returning a negative result [3], subjecting patients to increased healthcare costs and complication risks [4]. Furthermore, many patients (up to 16.0%) with a negative SLNB will experience tumor recurrence or die from their disease, most within the first 2 years following diagnosis [1, 5, 6, 7]. Of particular importance, patients who have early recurrence of their disease, particularly within the first year, have shorter overall survival and survival after recurrence than those who develop later recurrence [8, 9]. Although tools using only clinical and pathologic factors have attempted to identify patients who may potentially forego SLNB [10, 11], none have been shown to add benefit over performing SLNB for all eligible patients [12], been prospectively validated, or demonstrated that patients who forego SLNB based on the assessment tool are not harmed by recurrences. Additionally, because adjuvant therapy is now available for patients with stage IIB and IIC melanoma, a positive SLNB is not necessary to be eligible for adjuvant treatment. SLNB is associated with higher patient and healthcare‐related costs and a non‐negligible 11% complication rate [3, 4]. Thus, novel tools incorporating objective risk measures with clinical and pathological risk factors could help safely reduce unnecessary surgical procedures and associated healthcare costs and patient morbidity.

The 31‐gene expression profile (31‐GEP) test for CM is prospectively validated to identify patients at low (Class 1A), intermediate (Class 1B/2A), and high (Class 2B) risk of recurrence, metastasis, or death [13, 14]. Furthermore, the 31‐GEP is retrospectively and prospectively validated to identify patients with < 5% risk of SLN positivity when the 31‐GEP result is included in a patient's risk assessment with other factors when considering foregoing SLNB, and these low‐risk patients had high recurrence‐free survival (RFS), distant metastasis‐free survival, and melanoma‐specific survival rates [15, 16, 17]. Moreover, two recent studies demonstrated that patients tested with the 31‐GEP have higher survival rates than patients not tested with the 31‐GEP [14], and that patients with 31‐GEP testing have tumor recurrence detected earlier at a lower tumor burden with higher overall survival than patients without 31‐GEP testing [18], indicating that using the 31‐GEP as part of routine clinical care can improve patient outcomes.

The objective of the present study was to assess recurrence in patients who would have been recommended to forego SLNB based on low‐risk (Class 1A) 31‐GEP results. We assessed whether these patients experienced any recurrence, particularly early recurrences, regardless of whether an SLNB was performed or SLNB positivity.

## 2 Methods

The DECIDE (DecisionDx‐Melanoma Impact on Sentinel Lymph Node Biopsy Decisions and Clinical Outcomes) study is a prospective, multicenter study of patients with predominantly T1–T2 tumors being considered for an SLNB. The study assesses the performance of the 31‐GEP test in these SLNB‐eligible patients through evaluating long‐term follow‐up outcomes, and the effect of 31‐GEP testing on SLNB performance [16]. Briefly, patients diagnosed with invasive CM from May 2020 to May 2022 at 22 institutions (enrollment 89% by surgical oncologists, 8% by dermatologists, and 3% by medical oncologists) [16] for whom SLNB was being considered and who had the 31‐GEP test ordered for clinical reasons were included. The study was approved by the WCG Institutional Review Board (WCG‐IRB), and approval was obtained from the institutional IRBs at the participating centers. At Visit 1, patients who met inclusion criteria were provided informed consent and enrolled in the study. After reviewing all clinical data, including the 31‐GEP test results, the clinician proceeded with the decided treatment course. At protocol‐designated time points, information related to 31‐GEP test results, SLNB performance, and which clinical, pathological, molecular, and patient factors influenced the decision to perform an SLNB were recorded by designated site personnel into a secure, web‐based electronic case report form (eCRF). Subsequent follow‐up visits recorded recurrence outcomes through chart review or participant phone call at 6‐month intervals. Institutional review board approval was obtained at each participating institution prior to participant enrollment. Prior to the current analysis, 100% source data verification was completed to ensure data completeness and data management was completed for all participants to ensure data integrity. Missing ulceration status or mitotic rate information were considered absent or zero, respectively. Initial DECIDE study results confirmed the performance of the 31‐GEP to identify low‐risk patients (Class 1A) with < 5% likelihood of a positive SLNB and increased SLNB positivity rates in patients with a Class 1B/2A and Class 2B test result [16].

The original study by Yamamoto et al. [16] was powered to detect a significant reduction in SLNB performance compared with a group of patients who did not have the 31‐GEP as part of their clinical decision‐making surrounding SLNB, which met its primary endpoint. This subsequent report focuses on the early outcomes (RFS) of patients with T1a, T1b, T2a, or T2b tumors and a Class 1A 31‐GEP result (*n* = 131) who forego SLNB when integrating the 31‐GEP into clinical decision‐making to ensure patients considered low risk by the 31‐GEP (Class 1A) are not harmed by avoiding an SLNB. Although the 31‐GEP was recently integrated with clinicopathologic factors [17], the data included in the present study were obtained prior to the development of the integrated model; therefore, only the 31‐GEP Class result is shown here. RFS for patients with a Class 1A 31‐GEP result was estimated using Kaplan–Meier analysis.

## 3 Results

Patient demographics are shown in Table 1. There were no differences between patients who had an SLNB and those who did not for age (*p* = 0.492), ulceration status (*p* = 0.558), transected base (*p* = 0.416), or mitotic rate (*p* = 0.361). However, patients who received SLNB had slightly thicker tumors than those who did not (0.9 mm vs. 0.8 mm, *p* < 0.001). Follow‐up data were available for 94% (130/138) of the patients with a Class 1A result, with a median follow‐up time of 2.0 years (range 0.4–3.5 years). Of the patients with a Class 1A 31‐GEP result, 63 (48.5%) had an SLNB, with a 3.2% (2/63) SLN positivity rate (1 T1b and 1 T2a), and 67 (51.5%) did not undergo SLNB. No patient (0% [0/130]) with a 31‐GEP Class 1A result had a recurrence, regardless of SLN status (positive, negative, or not performed), at the time of the last follow‐up (100% 3‐year RFS).

**TABLE 1:** Patient demographics.

Factors	31‐GEP Class 1A (*n* = 130)	SLNB performed (*n* = 63)	SLNB unknown/not performed (*n* = 67)	*p*
Age (years), median (range)	65 (25–87)	65 (25–87)	65 (35–83)	0.492
Breslow thickness (mm), median (range)	0.9 (0.2–1.9)	0.9 (0.3–1.9)	0.8 (0.2–1.6)	< 0.001
Ulceration status, % (*n*)				
Absent/unknown	96.9% (126)	95.2% (60)	98.5% (66)	0.558
Present	3.1% (4)	4.8% (3)	1.5% (1)	
Transected base, % (*n*)				
No	41.5% (54)	46.0% (29)	37.3% (25)	0.416
Yes	40.0% (52)	39.7% (25)	40.3% (27)	
Unknown	18.5% (24)	14.3% (9)	22.4% (15)	
Mitotic rate (1/mm^2^), median (range)	1 (0–10)	1 (0–9)	1 (0–10)	0.361
SLN status				
Negative	46.9% (61)	96.8% (61)	—	—
Positive	1.5% (2)	3.2% (2)	—	
Not performed	51.5% (67)	—	100% (67)	
T stage				
T1a	33.1% (43)	17.5% (11)	47.8% (32)	< 0.001
T1b	43.1% (56)	47.6% (30)	38.8% (26)	
T2a	22.3% (29)	31.7% (20)	13.4% (9)	
T2b	1.5% (2)	3.2% (2)	0% (0)	
Recurrence, % (*n*)	0% (0/130)	0% (0)	0% (0)	
Median follow‐up time, years (range)	2.0 (0.4–3.5)	2.0 (0.4–3.0)	2.0 (0.5–3.5)	0.025

## 4 Discussion

Because some patients with a negative SLNB will still experience a recurrence or die from their disease [1], it is essential that tools attempting to identify patients with low risk of SLN positivity be evaluated for accuracy to predict the risk of disease progression to ensure the patient is not harmed by foregoing SLNB. The 31‐GEP is the only prospectively validated test to identify patients with CM at low risk of SLN positivity and low or high risk of tumor recurrence, metastasis, or death [13, 16]. Previous studies have determined that the 31‐GEP can better identify which patients can safely forego SLNB than either T stage or other GEP tests [19]. This current study is clinically significant as it is the first to demonstrate that using a GEP test for CM can help safely guide patients away from SLNB, as there were no early recurrences in the low‐risk (Class 1A) patient population, regardless of SLN status or whether SLNB was performed. Importantly, these results were obtained when the 31‐GEP was actively considered in combination with clinicopathological factors in the decision to avoid or perform an SLNB. Although not assessed in the current study, patients with a Class 1B/2A or Class 2B result have been shown to have > 5% risk of SLN positivity and increased risk of tumor recurrence compared to those with a Class 1A result [15]. Therefore, although patients with a Class 1A result can consider avoiding SLNB and have reduced management intensity, patients with a non‐Class 1A result should be considered for SLNB and increased management intensity.

Current guidelines suggest that patients with T1 tumors have a 5%–10% population‐estimated risk of SLN positivity (T1b and certain T1a with high‐risk features) and may choose to consider SLNB [20]. Weitemeyer et al. [21] showed that the change from the American Joint Committee on Cancer (AJCC) 7th edition to the 8th edition resulted in more SLNBs performed in patients with T1b tumors, though with a lower positivity rate. Multiple other studies demonstrate that SLNBs performed in patients with T1a and T1b tumors have high node‐negativity rates [22, 23, 24], with one study at primarily surgical centers showing that 66% of patients with a 5%–10% risk of SLN positivity had an SLNB performed [17]. SLNB can be costly, adding significant cost to the healthcare system, especially because most SLNBs (88%) are negative [3, 4]. A recent health economic impact study, which included using the 31‐GEP to guide SLNB decisions, found that including the 31‐GEP with AJCC staging, compared to staging alone, for SLNB management decisions resulted in a healthcare payer savings of over $500,000 per 1,000,000 plan members [25]. As demonstrated by Yamamoto et al., incorporating the 31‐GEP into clinical decision‐making regarding SLNB can significantly reduce the number of unnecessary SLNBs performed, thus reducing costs to the patient and healthcare system. Furthermore, the current study demonstrates that patients who forego SLNB based on the 31‐GEP result do so safely, as no early recurrences were noted at the time of last follow‐up. Although other biomarker tests are under investigation, they are indicated only for SLNB risk prediction [26] or risk of recurrence [27], whereas the 31‐GEP is indicated for both SLNB risk prediction and risk of recurrence stratification, as demonstrated in the current study. Furthermore, previous studies have determined that the 31‐GEP can better identify which patients can safely forego SLNB than either NCCN guidelines, the CP‐GEP, or other nomograms [12, 19]. Using the 31‐GEP in this population has the potential to significantly reduce the number of unnecessary SLNBs performed, while not adversely affecting RFS rates when compared with the use of other GEP tests or prediction nomograms, which have no clinical utility data and have not shown a benefit in patients with T1b tumors [12, 26, 28].

**FIGURE 1:**
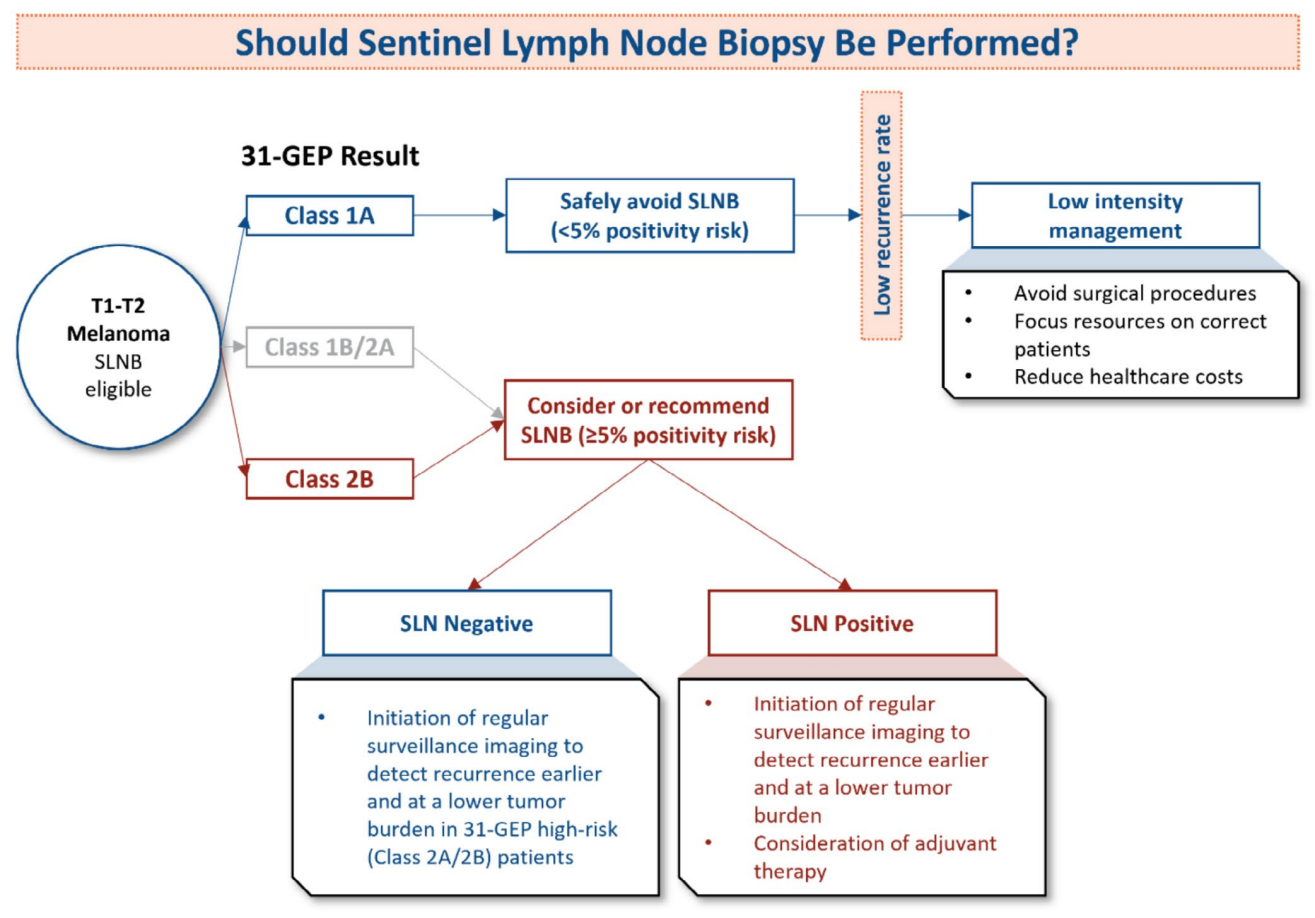
Flow chart incorporating 31‐GEP testing into sentinel lymph node biopsy and clinical management decisions for patients with T1–T2 cutaneous melanoma tumors.

These and other data suggest that the 31‐GEP provides valuable prognostic information in multiple steps of the patient's clinical course. First, using the 31‐GEP to guide SLNB decisions can reduce the number of SLNBs by at least 29%, with a low risk of recurrence [16]. Second, in patients with a negative SLNB, the 31‐GEP identifies patients at high risk of tumor recurrence (Class 2A and 2B) who should be followed more closely so that recurrence can be detected and treated earlier, as described previously (Figure 1) [18, 29].

Limitations of the study include the relatively small number of patients and low number of patients who had an SLNB to assess; however, the multicenter prospective study design increases the generalizability of the results to a wider population. Additionally, follow‐up time is limited to a median of 2 years; however, monitoring of patients is ongoing and prior studies have shown that the median time to recurrence in early‐stage melanoma is less than 2 years [8, 30], consistent with previously reported prospective data of the 31‐GEP indicating early‐stage patients with a Class 1A result who experience recurrence are likely to experience it within 2 years [13]. These data provide evidence that, in the current study, providers are using the 31‐GEP to select the correct patients with a Class 1A result who can safely avoid SLNB, and no recurrences were detected at a median follow‐up of 2 years [31, 32]. Understanding how the 31‐GEP can reduce healthcare‐related costs is of critical importance for patients and the healthcare system, and tools that can reduce costs should be studied.

## 5 Conclusions

The current study demonstrates that patients with a Class 1A result may safely forgo the SLNB procedure. No patients had a recurrence within the study period, regardless of SLN status, supporting the clinical value of the 31‐GEP test in guiding patient care.

## Author Contributions

**J. Michael Guenther:** investigation (equal), resources (equal), writing – review and editing (supporting). **Andrew Ward:** investigation (equal), resources (equal), writing – review and editing (supporting). **Brian J. Martin:** conceptualization (equal), formal analysis (equal), methodology (equal), validation (equal), visualization (equal), writing – original draft (equal), writing – review and editing (equal). **Mark Cripe:** investigation (equal), resources (equal), writing – review and editing (supporting). **Timothy Beard:** investigation (equal), resources (equal), writing – review and editing (supporting). **Oliver Wisco:** investigation (equal), resources (equal), writing – review and editing (supporting). **Rohit Sharma:** investigation (equal), resources (equal), writing – review and editing (supporting). **Stanley P. Leong:** investigation (equal), resources (equal), writing – review and editing (supporting). **Richard Essner:** investigation (equal), resources (equal), writing – review and editing (supporting). **Joseph I. Clark:** investigation (equal), resources (equal), writing – review and editing (supporting). **John Hamner:** investigation (equal), resources (equal), writing – review and editing (supporting). **Brenda Sickle‐Santanello:** investigation (equal), resources (equal), writing – review and editing (supporting). **Maki Yamamoto:** investigation (equal), resources (equal), writing – review and editing (supporting).

## Ethics Statement

The study was approved by the WCG Institutional Review Board (WCG‐IRB), and approval was obtained from the institutional IRBs at the participating centers.

## Conflicts of Interest

This study was funded by Castle Biosciences Inc. B.J.M. is an employee and stock/options holder at Castle Biosciences Inc. J.M.G. and R.S. are on the Speaker's bureau for Castle Biosciences. Additional authors have no conflicts to declare.

## Data Availability

Research data are not shared.
